# Microstructure and Microhardness Research of Steel 304 After Forming Partially Regular Reliefs by Ball Burnishing Operation

**DOI:** 10.3390/ma18071565

**Published:** 2025-03-30

**Authors:** Stoyan Dimitrov Slavov, Diyan Minkov Dimitrov, Desislava Yordanova Mincheva, Volodymyr Dzyura, Pavlo Maruschak, Volodymyr Semehen

**Affiliations:** 1 Department of Manufacturing Technologies and Machine Tools, Technical University of Varna, Studentska Str. 1, 9010 Varna, Bulgaria; sdslavov@tu-varna.bg; 2 Department of Mechanics and Machine Elements, Technical University of Varna, Studentska Str. 1, 9010 Varna, Bulgaria; dm_dimitrov@tu-varna.bg; 3 Department of Materials Science and Technology, Technical University of Varna, Studentska Str. 1, 9010 Varna, Bulgaria; dmincheva@tu-varna.bg; 4 Department of Motor Vehicles, Ternopil Ivan Puluj National Technical University, 46001 Ternopil, Ukraine; volodymyrdzyura@gmail.com (V.D.); semegen5@ukr.net (V.S.)

**Keywords:** surface microhardness parameters, microstructure, partially regular relief, ball burnishing, ANOVA, regression analyses

## Abstract

The influence of regular relief formation modes on the microhardness of the formed groove surface near the apex at the bottom of the groove has been studied. It has been established that the rate of plastic deformation, expressed as the feed rate of the deforming element, has a significant impact on the plastic deformation mechanism, and the microstructure of the formed subsurface layer, as well as on the microhardness of the groove surface. The influence of the type of partially regular reliefs on the degree of plastic deformation was also investigated. It was found that the third type of partially regular relief, which has the highest groove density, provides higher microhardness values than the first and second types. This is explained by the significantly greater density of these type of partially regular relief grooves, which exert a mutual strengthening effect on the surface during formation. The experimental study conducted enabled the derivation of regression equations describing the influence of the feed rate of the deforming element and the type of partially regular relief created on the surface microhardness beneath the lateral ridges and the bottoms of the plastically deformed traces.

## 1. Introduction

The study of surfaces with different types of regular microreliefs is primarily aimed at determining the influence of technological factors on the physical and mechanical properties of the surface to improve its durability [1,2,3] and wear resistance [4,5].

The main patterns of changes in the physical and mechanical properties of metals after the formation of regular and partially regular so-called “microreliefs” are summarized in [6]. It has been established that the surface ball burnishing process with the formation of a regular microrelief affects the magnitude of compressive stresses. Ground samples with a fatigue strength limit of σ_−1_ = 255 MPa were studied. The application of regular microreliefs increased their fatigue strength limit to 334 MPa, which is a 20% increase. To determine the influence of compressive stress, the samples with a regular microrelief were subjected to heat treatment, namely, tempering in argon at 650 °C for 1.5 h. As a result, the fatigue strength limit decreased to 285 MPa, i.e., an 11% reduction. Thus, although heat treatment reduces the strength limit, it ensures a uniform distribution of physical and mechanical properties across the entire surface with a regular microrelief.

An increase in surface hardness after laser texturing was identified in [7]. The authors investigated the effect of laser surface hardening and ultrasonic impact treatment on the surface’s roughness and microhardness of AISI D2 high-chromium tool steel. The study concluded that analyzing the hardness magnitude, depth, and width of the laser-hardened area allowed for selecting the optimal laser surface hardening (LSH) regime, which provided a ~150% increase in hardness due to transformation hardening with a minimal reduction in surface roughness. The optimal ultrasonic impact treatment (UIT) regime was determined based on the lowest achieved surface roughness. The optimal UIT led to severe plastic deformation, reducing surface roughness by ~80% and increasing hardness by ~50%.

The creation of an entirely new regular microrelief on the surface of a cylindrical shaft alters its operational properties [8]. The microrelief was applied using a vibrating tool with a radius of R = 2 mm under a load of P = 550 N. As a result of treatment, the surface roughness based on the arithmetic mean deviation was Ra = 0.63 µm, which is 7.6 times lower than the initial value. Experimental studies confirmed that different regimes of treatment significantly alter surface roughness, hardness, and microhardness. It was found that comprehensive treatment technology increased surface hardness by 1.2 to 1.6 times compared to the base metal hardness.

The microhardness of textured surfaces with grooves of different shapes, created using a laser method, was analyzed in [9]. A comparison was conducted between surfaces without the formed microstructures, surfaces with non-intersecting straight grooves, and surfaces with straight grooves intersecting at right angles. It was established that a surface with intersecting grooves provided a hardness 100 units HV higher than a surface without formed regular microstructures. Additionally, this surface exhibited a hardness 50 units HV higher than that of non-intersecting grooves. This indicates that a greater number of deformation-affected locations results in higher surface hardness.

The influence of the formed microrelief and its shape on the friction coefficient and surface wear of the Ti-6Al-4V alloy is described in [10]. The study also generalized the impact of surface texturing on its microhardness. Several configurations of the microrelief elements, in the form of hemispherical dimples, were analyzed for their effect on microhardness. It was found that a microgrid configuration provided the highest microhardness, reaching 400–405 units HV, which is 35–40 units HV higher than other microrelief configurations. Additionally, this configuration also ensured the lowest friction coefficient. Another important finding was that an increase in the relative area of the microrelief beyond 51% significantly reduced the friction coefficient due to the decrease in the contact surface area.

A new technological process has been considered, which additionally includes turning operations with or without cryogenic pre-cooling of the workpiece and surface plastic deformation operations by ball burnishing [11]. The method for performing cryogenic pre-cooling, rigid turning, and ball polishing to achieve satisfactory surface integrity and operational properties has been described. Surface roughness was evaluated in 2D and 3D dimensions, microstructure changes were analyzed using SEM/BSE methods, and microhardness distribution was assessed. Some geometric, mechanical, and physical improvements in the properties of the surface layer were identified.

During surface plastic deformation using a deforming element, a gradient of hardness surface is formed across the depth of the surface. This gradient depends on the treatment regime parameters and the geometric characteristics of the deforming element. The dependence of microhardness on the distance from the surface subjected to surface plastic deformation is described in [12]. It was found that its value initially increases in the subsurface layer before gradually decreasing with increasing depth.

The effect of burnishing parameters on microhardness changes was investigated in [13]. It was established that burnishing cylindrical surfaces with a spherical burnishing tool and increasing the feed rate within the range of small values (0.01–0.05 mm/rev) increases microhardness by 12%. It was found that the feed rate had the most significant impact on microhardness changes, whereas workpiece rotational speed had the least influence among all studied parameters.

Research results on the microhardness of surface layers on cylindrical surfaces with a formed regular microrelief using vibratory burnishing with a spherical deforming element under a deformation force of 350 N are known [14]. It was established that microhardness gradually decreases with increasing distance from the surface with the formed microrelief and stabilizes at approximately 0.32 mm depth.

An increase in the microhardness of the processed surface by 28% after burnishing with roller-type deforming elements compared to the initial state was found in [15]. It was determined that microhardness depended on the spindle speed of the machine tool, with values in the range of 1000–2500 rpm, causing a significant increase in surface microhardness. However, increasing the feed rate within the range of 200 to 450 mm/min led to a decrease in microhardness.

A contradictory result regarding the effect of the feed rate on microhardness changes was obtained in [16]. It was found that increasing the feed rate from 0.2 to 0.6 mm/rev for cylindrical surfaces led to a 10–15% increase in microhardness. Additionally, increasing the workpiece rotational speed from 355 to 710 rpm also resulted in an 8–10% increase in microhardness.

In [17], the dynamic plastic deformation of 304L stainless steel was investigated. A significant influence of the microstructure of the formed surface on the rate of its deformation was established. An improvement in mechanical properties due to the increase in dislocations α’-martensite phases was also noted.

In [18], the effect of different thickness reductions by cold-rolling on the microstructure and the mechanical properties of AISI 304 austenitic stainless steel were investigated. The results of the research indicated that the formation of strain-induced martensite resulted in a significant strengthening of the steel.

Thus, summarizing the effect of plastic deformation on microhardness changes, it should be noted that microhardness increases due to the strengthening of surface and subsurface layers. However, the effect of the feed rate of the deforming element on surface microhardness requires further research, as the literature presents contradictory findings. Additionally, the change in microhardness of the surface layer around the formed groove and at various distances from the groove requires further investigation. This will enable us to determine the optimal relative placement of microrelief grooves on the surface of the processed parts.

The main objective of this study is to assess the influence of some ball-burnishing operation regime parameters and three types of partially regular relief formatted onto AISI 304 steel flat specimens on the microhardness in the depth of the surface layer and to provide a physical–mechanical interpretation of these effects. The second objective is to derive appropriate mathematical models, which give the relation between the regime parameter “feedrate”, and the type of the researched partially regular reliefs and resulting microhardness beneath the lateral ridges and bottoms of the plastically deformed traces after ball burnishing operation applying.

Due to the obvious discrepancy between the originally introduced terms “regular-microreliefs” and “partially-regular-microreliefs” in [6] and the actual sizes of the groove’s patterns that are obtained, in the present work, the terms “regular relief” (RR) and “partially regular relief” (PRR) will be used instead of the original ones. We believe that using these proposed terms could be more appropriate considering the actual scales of the obtained plastically deformed groove’s patterns, which are about 10^3^ times larger than the micrometer unit scale and can reach up to several millimeters.

## 2. Materials and Methods

### 2.1. Preparing the Test Specimens

**Figure 1:**
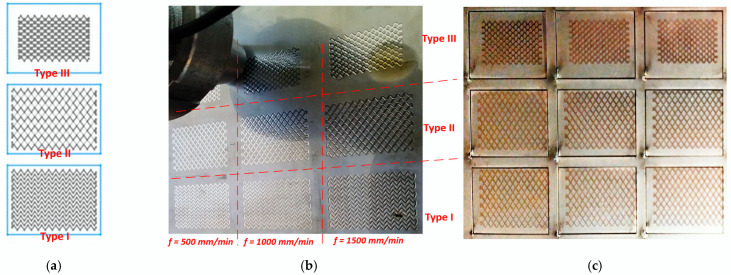
Diagram of the three types of partial regular reliefs (**a**); forming the PRR onto the top surface of the plate using BB process (**b**); the cut-out smaller specimens from the plate for microhardness measurements and structure analyses (**c**).

According to the main goal of the present research, three types of partial RR (PRR) are investigated, which are obtained by applying ball burnishing process on the top surface of a prismatic plate, made of AISI 304 stainless steel cold-rolled sheet. The types of PRR differ by how plastically deformed traces are patterned onto the specimen’s surface (see Figure 1a–c). Depending on it, they can be classified as follows [6]:

(a) Pattern with traces that do not touch each other (type I);

(b) Pattern with traces that touch each other (type II);

(c) Pattern with traces that are crossing between each other (type III).

The three types of PRR with three different feed rates are formed onto a single plate using HAAS (Philadelphia, PA, USA) TM-1 CNC milling machine and specially developed deforming tool [19] (see Figure 1b). After that, the plate is cut out into smaller size specimens with dimensions of 30 × 40 × 4 mm (see Figure 1c), using a fiber laser CNC-machine, type GN NCF 3015 (Wuhan Gnlaser Equipment Manufacturing CO., LTD, Wuhan, China). An austenitic stainless-steel cold-rolled plate (grade AISI 304) with 4 mm thickness was utilized, which has the mechanical properties shown in Table 1, according to the certificate issued by the sheets’ manufacturer. The chemical composition of the material is measured by using a model S1 Sorter XRF analyzer (Bruker Corp., Billerica, MA, USA). The resulting chemical composition in wt% is given in Table 2.

To keep the surface with PRR while cutting the pieces for metallography cross-sections, a thin copper film coating was applied to the upper surface of the specimens, by the “brush plating” technology. The laying of the coating is carried out as follows: The sample is caught in a handle that is connected to the negative pole of the power source. The graphite electrode wrapped in a textile braid connects to the positive pole of the power source. With the current source on, the electrode is immersed in the appropriate solution and by rapid movements, performs the application of the coating on a local part of the specimen surface.

One of the lateral surfaces of the kerfs, which pass normally through the deformed traces as sections, is additionally mechanically processed using a metallographic grinding and polishing machine, type MoPao 260E (Opassy Industry Group Co., Ltd., Dongguan, China), for the purpose of making suitable specimens for metallographic analyses and microhardness measurements and removing the material layer that is changed after the laser cutting operation. The cut-out smaller metallographic specimens are subjected to thermo-hardening plastic pressing first, using an XQ-2B metallographic specimen inlaying machine, and then to further polishing to reach the needed surface smoothness for microhardness measuring. For further microstructure analysis, the cross-sections are etched with a reagent suitable for austenitic steel composed of a 1:3 volume ratio mixture of concentrated nitric acid (HNO₃) and concentrated hydrochloric acid (HCl).

### 2.2. Initial Microstructure and Microhardness Measurement of the PRR

To prepare a substantiated experimental plan, a preliminary microstructure and microhardness measurements of specimens that have PRR from Type I were conducted.

The microhardness of the deformed structure was measured by the Vickers method with a load of 50 g, according to ISO6507-1 [20]. An HVS-1000 Digital Micro Hardness Tester (Opassy Industry Group Co., Ltd., Dongguan, China) was used.

**Figure 2:**
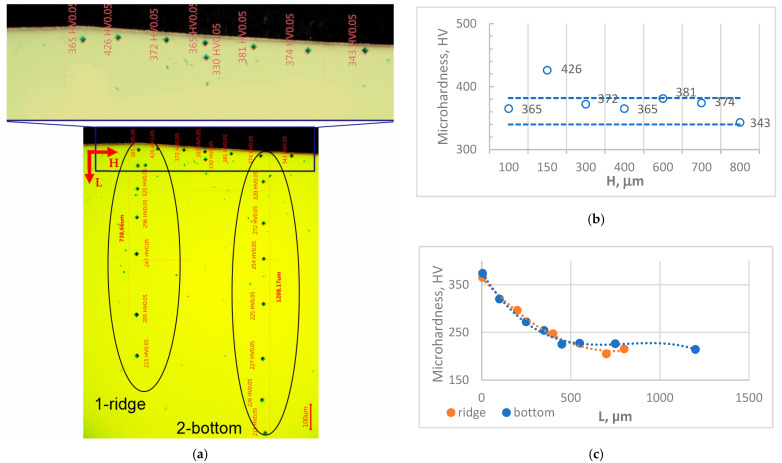
(**a**) The cross-section of measured microhardness for PRR from type I; (**b**) diagrams of changing the microhardness in the depth of the material beneath the traces ridge (1) and bottom (2); (**c**) diagram of the microhardness changing along the trace section.

The microhardness measurements in the depth of the surface layer and along the PMM trace section are shown in Figure 2a, and the diagrams of microhardness variation are shown in Figure 2b,c.

Measurements taken very close to the surface level are in the range of 340–380 HV_0.05_ and only one indentation is above 380, which can be local structure fluctuation due to the strain-induced martensitic area, as shown in Figure 2b. There is no tendency of an increase in the values going from the ridge to the bottom of the groove.

The lower indentation traces indicate the varying of microhardness values in depth. The summarized graph in Figure 2c shows a sharp decrease in values to the depth of about 500 µm similar for both traces. Deeper, the bottom trace is higher than the ridge trace with microhardness value 225 HV_0.05._ This trace reaches the steel microhardness 215 HV_0.05_ below a 1000 µm depth. It can be summarized that even the hardness near the surface of both traces is similar, and the difference between the measured microhardness near the trace’s bottoms and near the ridges around 30–50 HV_0.05_ is also observed, which confirms the distribution of the degree of plastic deformation during the BB finishing operations derived in their classical application (i.e., smoothing the surface) [21].

### 2.3. Experimental Design Description

The BB operations are conducted with three different feed rates of the ball tool: f = 500, 1000, and 1500, mm/min for every of the three types of PRRs. Therefore, the main factors of the current experimental investigation are the three types of patterns and three feed rates of the BB operation. The deforming force F, N is kept constant for all formed PRRs and has a magnitude of 400 N.

**Figure 3:**
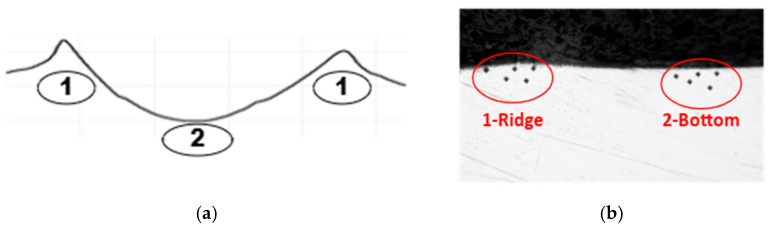
Diagram of the measurement areas of the PRR’s traces cross-sections (**a**); image of microhardness measurements at the ridges ${HV}_{0.01}^{R}$ (area 1) and bottoms $\;{HV}_{0.01}^{B}$ (area 2) of PRR that are of Type I to III (**b**).

After processing the PRRs, the test specimens are obtained, as described in Section 2.2. Microhardness measuring is performed in two main areas: near the top of the plastically deformed trace ridges (area 1), and near the bottom of the traces (area 2) (see Figure 3a).

Because the HV measurement repetitions should not be too close to each other to avoid interference between them, and, at the same time, the area of measurements at the ridges section is comparatively narrow, the applied force of the test device indenter was chosen to be 10 g instead of 50 g. Therefore, the outcome parameters from the experimental investigation are ${HV}_{0.01}^{R}$ and ${HV}_{0.01}^{B}$. To compensate for the microstructure-dependent hardness values and so-called “indentation size effect” [22] on lower-load indents, measurements were carried out in five-time repetitions for every combination of the design factors levels to obtain more reliable results for ${HV}_{0.01}^{R}$ and ${HV}_{0.01}^{B}$. The experimental design and the results obtained for hardness measurements are shown in Table 3.

## 3. Results

### 3.1. Results for Microhardness Change

According to modern concepts of deformation processes, the application of RR caused localized plastic deformations on the surface of an austenitic stainless-steel plate (grade AISI 304). From a physical perspective, the plastic deformation process led to the accumulation of volumetric damage in the surface layer, where defects and damage were introduced into the steel’s microstructure. It was found that, for all types of PRRs studied, an increase in the feed rate, and consequently in the deformation speed, resulted in material strengthening at the micro level for all PRRs types investigated.

**Figure 4:**
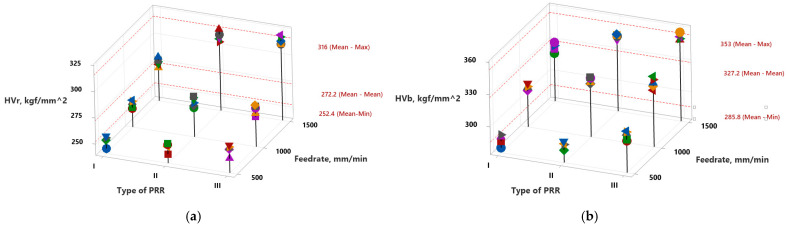
Three-dimensional scatter and trend plots that visualize HVs’ amendment, based on the values given in Table 2: (**a**) at the area of ridges of the PRRs; (**b**) beneath the bottoms of the PRRs’ plastically deformed traces.

This was confirmed by the increase in microhardness values of ${HV}_{0.01}^{R}$ and ${HV}_{0.01}^{B}$, as shown in Table 3 and the 3D scatter plots in Figure 4a,b. As can be seen, an overall increase in surface hardness occurred in comparison with the initial material hardness ${HV}_{0.01}=205$ (see Table 1).

### 3.2. Microstructure Analysis of the Deformed Surface Layer

The microstructure of the steel in its initial state consists predominantly of austenite grains, with some areas of strain-induced martensite due to rolling. During the formation of the regular micro-relief, the bottom of the groove remains smooth, free from macro- and micro-defects. The traces of the deformation tool’s movement are uniform, indicating the stability of the deformation effect on the material. At the bottom of the lubrication groove, a highly deformed microstructure is formed, characterized by a high dislocation density [23]. The strengthening capability of austenitic steel is higher than that of steels of other classes, as reflected in the distinctly plastically deformed layer. This layer is barely noticeable at a feed rate of *f_in.i_*. = 500 mm/min, distinctly pronounced at *f_in.i_*. = 1000 mm/min, and it exhibits clear boundaries with the base metal at *f_in.i_*. = 1000 mm/min. It is formed by the subdivision of austenite grains into finer elements, followed by the amorphization of the surface layer, making individual grains indistinguishable and developing strain-induced martensite.

**Figure 5:**
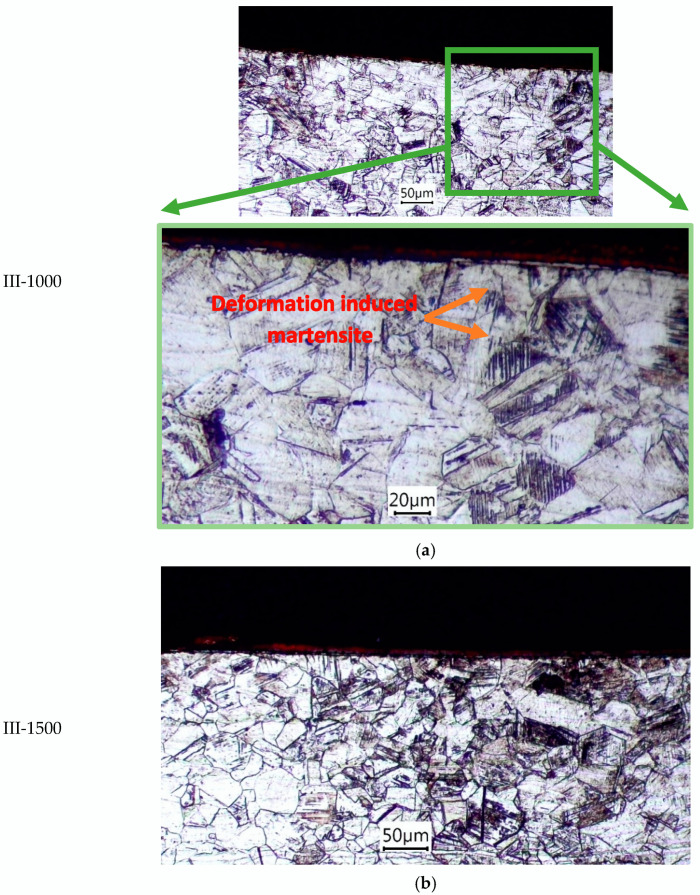
Optical micrographs of microstructures of austenitic stainless steel (grade AISI 304) obtained for different values of the feed rate, [mm/min]: (**a**) 1000; (**b**) 1500 for Type III of the relief.

The subsurface layers are formed through plastic deformation and displacement of austenite grains (see Figure 5). The deformation twin lines and areas transformed to strain-induced martensite are visible. This occurs because the groove profile is created by the plastic indentation of the metal both forward and laterally. Beyond the deformed surface, the microstructure remains almost unchanged.

With an increase in the feedrate, a greater deformation depth was observed. The maximum microhardness for the investigated processing conditions was similar, ranging from 314 to 377 HV_0.05_, indicating the maximum material strengthening under the given temperature–force processing conditions.

## 4. Derived Regressions Models and Its Statistical Analyses

Thus, the intensity of microhardness increases around PRRs formation area, depending on:

- The type and parameters of the deforming tool.
- The ball indentation force.
- The feed rate, as demonstrated above.

Therefore, structural and parametric optimization of existing technological modes, as well as the development of some new ones for the formation of an ordered PRR, is essential.

The results for ${HV}_{0.01}^{R}$ and ${HV}_{0.01}^{B}$ that are shown in Table 1 are used to analyze the impact of the type of PRR and feed rate factors of the ball burnishing operation on the basis of derived regressions equations and statistical analyses on it carried out by using Minitab 2022 (Minitab, LLC, State College, PA, USA) software [24]. They are as shown below.

### 4.1. Influence on the Microhardness Obtained at the Ridges Area of the PRRs Traces

The obtained coefficients of the main effects and interactions, along with the full regression equation, which is related to the impact of different types of PRRs and feed rates over the microhardness at the ridges ${HV}_{0.01}^{R}$, are as follows:

*HV^R^*_0.01_(*PRR*, *f*) = 275.467 − 12.267·*PRR_I_* + 3.733·*PRR_II_* + 8.533·*PRR_III_* − 19.067·*f*_500_ − 6.133·*f*_1000_ + 25.200·*f*_1500_ + 8.270·*PRR_I_·F*_500_ + 4.330·*PRR_I_·f*_1000_ − 12.600·*PRR_I_·f*_1500_ − 4.930·*PRR_II_·f*_500_ − 0.870·*PRR_II_·f*_1000_ + 5.800·*PRR_II_·f*_1500_ − 3.330·*PRR_III_·f*_500_ − 3.470·*PRR_III_·f*_1000_ + 6.800·*PRR_III_·f*_1500_ (1)

The statistical assessment of the regression Equation (1) shows a standard deviation of σ = 4.391, and an R-square = 96.8%, which means that the model has a very good fit against the experimentally obtained results. To assess the statistical significance of the regression predictors, using the null hypothesis theorem, an Analysis of Variance (ANOVA) was conducted. The results of it are shown in Table 4.

As seen from Table 4, all predictors and interaction between them from the regression model (1) are statistically significant due to the close-to-zero *p*-values calculated, which means that the null hypothesis theorem is rejected for all of them. Considering the percentages of contribution (see Table 4), the feed rate has a higher impact (71.74%), followed by the type of PRR (16.42%) and the interaction between them (8.63%) over parameter ${HV}_{0.01}^{R}$.

### 4.2. Influence on the Microhardness Obtained at the Bottom Area of the PRRs’ Traces

The full regression equation that describes the influence of PRRs’ types and feed rates over the microhardness at the bottom ${HV}_{0.01}^{B}$ is as follows:

*HV^B^*_0.01_(*PRR*, *f*) = 319.911 − 12.644·*PRR_I_* + 0.622·*PRR_II_* + 12.022·*PRR_III_* − 24.644·*f*_500_ + 4.222·*f*_1000_ + 20.422·*f*_1500_ + 3.180·*PRR_I_·F*_500_
*− 0.090*·*PRR_I_·f*_1000_ − 3.090·*PRR_I_·f*_1500_ − 4.890·*PRR_II_·f*_500_ + 2.440·*PRR_II_·f*_1000_ + + 2.440·*PRR_II_·f*_1500_ + 1.710·*PRR_III_·f*_500_ − 2.360·*PRR_III_·f*_1000_ + 0.640·*PRR_III_·f*_1500_ (2)

The regression Equation (2) has a standard deviation of σ = 3.551, and an R-square = 97.84%, which means that the model has a very good fit to the experimentally obtained results. To assess the statistical significance of the regression predictors, using the null hypothesis theorem, an Analysis of Variance (ANOVA) was conducted, and its results are shown in Table 5.

As seen from Table 5, all predictors and interaction between them from the regression model (2) are also statistically significant because the *p*-value statistics calculated for them are lower than 0.05 (i.e., 5%). This means that the null hypothesis theorem is rejected for all of them. The percentages of contribution in the model (see Table 5) are as follows: the feed rate has the highest impact (74.51%), followed by the type of PRR (21.79%). The interaction between them has contribution of just 1.53% over parameter ${HV}_{0.01}^{B}$ in model (2).

### 4.3. Main Effects and Interactions Diagrams

**Figure 6:**
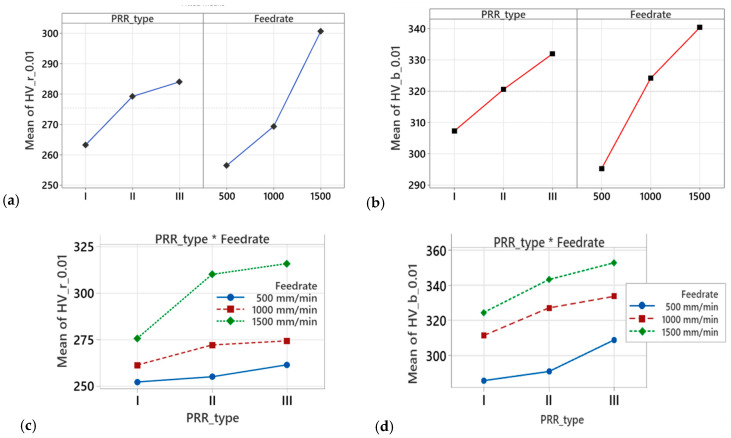
Main effects plot and interactions plot for: (**a**,**c**) microhardness HV_0.01_ at the ridges of PRRs; (**b**,**d**) microhardness HV_0.01_ at the bottoms of PRRs.

Based on the derived regression models (1) and (2), a graphical representation of the main effects and interactions of the regression models’ factors is shown in Figure 6.

The diagrams of main effects (see Figure 6a,b) show that there is an increase in HV_0.01_ for both areas of measurement—beneath the ridges and beneath the bottom of PRR traces, with an increase in the feed rate of the BB operation. A similar tendency occurs for different types of PRR, starting with PRR of Type I, where the traces do not touch each other, and reaching PRR of Type III, where they cross each other. As can be seen from the interaction plots shown in Figure 6c,d, the tendencies of the impact are very close to those observed for the main effects, both for microhardness HV_0.01_ at the grooves’ bottom and at the ridge areas.

## 5. Conclusions

Summarizing the research conducted, it can be stated that the type of PRR and the deformation speed significantly influence the microhardness variation in the subsurface layers of PRR’s grooves. As can be seen from the Min and Max mean values on the scatter plots (see Figure 4), this increase varies from 23.1% to 54.1% around the ridges (see pos. 1 in Figure 3a) and from 39,4% to 72.2% beneath the bottom of the plastically deformed traces (see pos. 2 in Figure 3a). It depends both on the BB operation regime parameter, feed rate and on the type of PPR formed onto the surface. In fact, the type of PRR essentially characterizes the groove density on the surface area and, consequently, determines their mutual influence on microhardness during formation. The highest microhardness (both at the edges and at the bottom of the grooves) was observed for Type III PRR, where the groove density was the highest, while the lowest microhardness was recorded for Type I grooves, where the density was the lowest.

Thus, understanding the influence of PRR type and deformation speed on surface microhardness allows for controlled variation of this parameter within 23.1 to 72.2%, depending on the selected PRR type and its formation speed (i.e., BB operation feed rate). The obtained results indicate the possibility of regulating the microstructural state of the bottom and edges of the ordered PRR grooves by adjusting the deformation intensity in the area under treatment. The primary influencing parameter is the feed rate, which controls the deformation speed and determines the structural-phase state and microhardness of the surface and subsurface layers of AISI 304 stainless steel.

Although the obtained regression models (see Equations (1) and (2)) are empirical in nature since they were derived for a specific study under certain conditions, the statistical analysis shows that they correctly describe the influence of the operating mode parameters on the resulting microhardness. They can be used to plan further studies to search for optimal settings of the operating mode parameters of the BB operation outside the limits of variation used in the present study, which will be the future work of our team.

## Figures and Tables

**Table 1:** Mechanical properties of austenitic stainless-steel AISI 304 cold-rolled sheet.

Mechanical Properties				
Material	Yield Strength, MPa	Ultimate Tensile Strength, MPa	Elongation A, %	HV_0.01_
AISI 304	372 ± 10	710 ± 12	31.2 ± 0.8	205 ± 12

**Table 2:** Chemical composition of austenitic stainless-steel AISI 304 cold-rolled sheet.

Material	Cr	Mn	Mo	Ni	Si	Fe
AISI 304	18.23	1.76	0.27	8.33	0.38	71.03

**Table 3:** Experimental design and results for microhardness values of ${HV}_{0.01}^{R}$ and ${HV}_{0.01}^{B}$.

NO.	Type of PRR	Feed Rate, *f_in.i_*. [mm/min]	${HV}_{0.01}^{R}$, [kgf/mm^2^]	$\mathrm{Mean}\;{HV}_{0.01}^{R}$, [kgf/mm^2^]	${HV}_{0.01}^{B}$, [kgf/mm^2^]	$\mathrm{Mean}\;{HV}_{0.01}^{B}$, [kgf/mm^2^]
1	I	500	244	252.4	289	285.8
2	I	500	256		279	
3	I	500	254		285	
4	I	500	256		291	
5	I	500	252		285	
6	I	1000	265	261.4	311	311.4
7	I	1000	263		312	
8	I	1000	257		308	
9	I	1000	258		315	
10	I	1000	264		311	
11	I	1500	278	275.8	319	324.6
12	I	1500	281		326	
13	I	1500	274		329	
14	I	1500	271		326	
15	I	1500	275		323	
16	II	500	248	255.2	292	291.0
17	II	500	258		286	
18	II	500	257		291	
19	II	500	258		292	
20	II	500	255		294	
21	II	1000	278	272.2	324	327.2
22	II	1000	275		328	
23	II	1000	272		330	
24	II	1000	267		325	
25	II	1000	269		329	
26	II	1500	312	310.2	341	343.4
27	II	1500	317		341	
28	II	1500	309		345	
29	II	1500	305		344	
30	II	1500	308		346	
31	III	500	253	261.6	312	309.0
32	III	500	266		313	
33	III	500	262		304	
34	III	500	264		305	
35	III	500	263		311	
36	III	1000	268	274.4	334	333.8
37	III	1000	278		340	
38	III	1000	276		337	
39	III	1000	271		327	
40	III	1000	279		331	
41	III	1500	315	316.0	350	353.0
42	III	1500	321		357	
43	III	1500	312		354	
44	III	1500	319		351	
45	III	1500	313		353	

**Table 4:** Results from ANOVA analysis of the regression model (1).

Source	DF	Seq SS	Contribution	Adj SS	Adj MS	F-Value	*p*-Value
Model	8	20,971.2	96.80%	20,971.2	2621.40	135.98	0.000
Linear	4	19,101.3	88.17%	19,101.3	4775.33	247.71	0.000
PRR_type	2	3558.4	16.42%	3558.4	1779.20	92.29	0.000
Feed rate	2	15,542.9	71.74%	15,542.9	7771.47	403.13	0.000
Two-Way Interactions	4	1869.9	8.63%	1869.9	467.47	24.25	0.000
PRR_type · Feed rate	4	1869.9	8.63%	1869.9	467.47	24.25	0.000
Error	36	694.0	3.20%	694.0	19.28		
Total	44	21,665.2	100.00%				

**Table 5:** Results from ANOVA analysis of the regression model (2).

**Source**	**DF**	**Seq SS**	**Contribution**	**Adj SS**	**Adj MS**	**F-Value**	*p*-Value
Model	8	20,527.6	97.84%	20,527.6	2565.96	203.47	0.000
Linear	4	20,205.7	96.30%	20,205.7	5051.42	400.55	0.000
PRR_type	2	4572.0	21.79%	4572.0	2286.02	181.27	0.000
Feed rate	2	15,633.6	74.51%	15,633.6	7816.82	619.84	0.000
Two-Way Interactions	4	322.0	1.53%	322.0	80.49	6.38	0.001
PRR_type · Feed rate	4	322.0	1.53%	322.0	80.49	6.38	0.001
Error	36	454.0	2.16%	454.0	12.61		
Total	44	20,981.6	100.00%				

## Data Availability

The original contributions presented in this study are included in the article. Further inquiries can be directed at the corresponding author.
